# An Updated Review of Bioactive Peptides from Mushrooms in a Well-Defined Molecular Weight Range

**DOI:** 10.3390/toxins14020084

**Published:** 2022-01-22

**Authors:** Nicola Landi, Angela Clemente, Paolo V. Pedone, Sara Ragucci, Antimo Di Maro

**Affiliations:** Department of Environmental, Biological and Pharmaceutical Sciences and Technologies (DiSTABiF), University of Campania ‘Luigi Vanvitelli’, Via Vivaldi 43, 81100 Caserta, Italy; nicola.landi@unicampania.it (N.L.); angela.clemente@unicampania.it (A.C.); paolovincenzo.pedone@unicampania.it (P.V.P.)

**Keywords:** bioactive peptides, fungi, mushrooms, molecular weight

## Abstract

Here, we report the current status of the bioactive peptides isolated and characterized from mushrooms during the last 20 years, considering ‘peptide’ a succession from to 2 to 100 amino acid residues. According to this accepted biochemical definition, we adopt ~10 kDa as the upper limit of molecular weight for a peptide. In light of this, a careful revision of data reported in the literature was carried out. The search revealed that in the works describing the characterization of bioactive peptides from mushrooms, not all the peptides have been correctly classified according to their molecular weight, considering that some fungal proteins (>10 kDa MW) have been improperly classified as ‘peptides’. Moreover, the biological action of each of these peptides, the principles of their isolation as well as the source/mushroom species were summarized. Finally, this review highlighted that these peptides possess antihypertensive, antifungal, antibiotic and antimicrobial, anticancer, antiviral, antioxidant and ACE inhibitory properties.

## 1. Introduction

Mushrooms are one of the most important natural sources of bioactive compounds for the presence of numerous products with therapeutic properties. Commonly used in traditional Chinese medicine for centuries, mushrooms are rich in natural antioxidative, antitumor, antiviral, antimicrobial and immunomodulatory agents, with medicinal effects proven by researchers [1,2,3]. Indeed, mushrooms have been demonstrated to be able to produce a great number of ‘potential drugs’, such as polysaccharides, phenolics, sterols, proteins and peptides responsible for the therapeutic effects attributed to this species [4,5,6]. In this regard, a peptide able to exert physiological effects is considered a bioactive peptide (BP) [7], and isolated small protein fragments with positive effects on human health can be considered BPs. Indeed, differing from proteins, peptides are completely absorbed at the intestinal level, causing local effects or reaching the target cells in intact forms through the cardiovascular system [8]. BPs are more stable and less toxic and possess a higher affinity for tissues. Thanks to their characteristics, the use of BPs as functional foods or in medicine is considered helpful in avoiding the side effects usually related to synthetic compounds [9]. They represent a very heterogeneous class of compounds with a molecular weight lower than proteins, differing in their structure and biological function and of great interest to researchers. Peptides consist of at least two amino acids in which the carboxyl group of the first L-alpha-amino acid is linked to the amino group of the subsequent one by a covalent chemical bond, also known as secondary amide or peptide bond (-CO-NH-) through a condensation reaction [10]. Amino acid composition and the length of the chain are important molecular characteristics of peptides. It is conventional to write the structure of a peptide with a free amino acid group at the left end and a free carboxyl group at the right end. Peptides are named by regarding them as acylated derivatives of the last amino acid residue (e.g., Gly-Ala is named glycyl-alanine); the names of acylating residues are then derived from the trivial name of the amino acid by replacing the ending, usually -ine or -yl. A PubMed database search (https://pubmed.ncbi.nlm.nih.gov/; accessed on 7 September 2021) using the keywords ‘mushroom peptides’ reveals more than 2500 published papers, most of which were published in the last 20 years. However, although there are many reviews in the literature on peptides from mushrooms/fungi, either there is not a proper classification of fungal BPs or the confusion on their classification is recurrent, mainly due to the fact that some authors also include in their fungal peptide classification polypeptide chains with a molecular weight greater than 10 kDa, and in some cases even greater than 20 kDa. For example, in 2015, Poompouang and Suksomtip isolated and characterized a novel antifungal peptide from fruiting bodies of the edible basidyomicetes mushroom *Lentinus squarrosulus* Mont. However, the peptide reported has a molecular weight of about 17 kDa, according to SDS-PAGE analysis [11]. On the other hand, Wang et al. avoid the term protein or peptide, preferring to use the generic term of ‘polypeptide’ for referring to alveolarin (28 kDa from gel filtration) from the wild mushroom *Polyporus alveolaris* [12].

According to the definition by the International Union of Biochemistry and Molecular Biology, a peptide is any compound produced by an amide formation between a carboxyl group of one amino acid and an amino group of another, in which the amide bonds in peptides may be called peptide bonds [13]. However, the distinction between a polypeptide and a protein is imprecise and largely academic. Peptides can be classified according to the number of amino acid residues, and it is well-established that a succession of 10 or fewer amino acids is considered an oligopeptide. A certain confusion occurs for polypeptides. Indeed, some authors define these as peptide molecules consisting of 10 to 50 amino acids (~1–5 kDa), and according to this definition, proteins are molecules with more than 50 amino acids [14]. Conversely, following the academic definition, other authors adopted an upper limit of 10 kDa for the molecular weight of a polypeptide, thus defining as a peptide a molecule composed of up to about 100 amino acids [15]. According to this last definition, the present work represents an updated review of all BPs isolated from mushrooms in the last 20 years, limiting our analysis to the molecules, which are in the correct molecular weight range while excluding the ones with higher molecular weight, which should be classified as proteins.

## 2. Physico-Chemical Properties

Beside the structural differences, BPs share some structural features, such as length (from 2 to 20 amino acid residues) and a prevalence of hydrophobic amino acid residues [16]. The acid–basic characteristics of amino acids are transferred to the peptides in which they are embedded. Specifically, the acid–base behavior of the peptides is determined by: (i) the free α-amino group of the N-terminal residue (pK_a_ slightly lower than that of the corresponding free amino acid), (ii) the free α-carboxylic group of the C-terminal residue (pK_a_ slightly higher than that of the corresponding free amino acid) and (iii) the ionizable groups of side chains of the residues in the chain. The reactivity, including acylation and esterification reactions, of amino and carboxyl terminal groups of a peptide is substantially similar to that of free amino acids. In particular, the amino group reactivity with some reagents, such as ninhydrin and *o*-phtaldialdehyde, is used for peptide detection and quantification [17]. In the presence of heating treatment and carbonyl compounds such as sugars, some peptides can be modified by the Maillard reaction, with the formation of a series of melanoidin pigments that contribute to the development of flavors, aromas and colors desired or not in foods [18]. Finally, protein hydrolysis into peptides during food processing (such as cooking or fermentation) and storage contributes to food’s rheological properties [19].

## 3. Functional Properties

**Figure 1:**
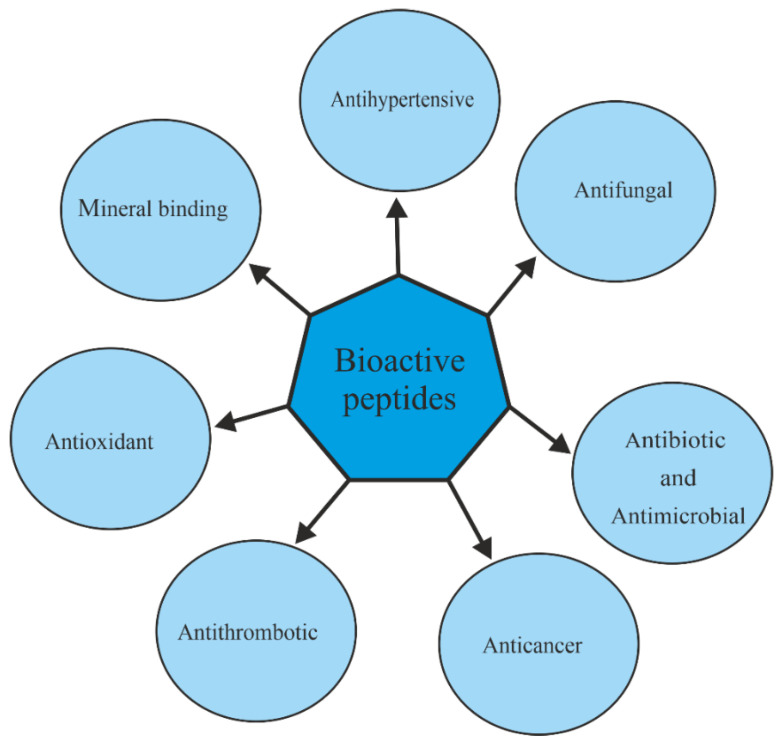
Schematic representation of the most well-known BP properties.

A number of BPs derived from proteins remain inactive, acquiring their specific function only upon release (enzymatic hydrolysis) from parental-derived proteins. Besides their high nutritional value due to the presence of essential amino acids, BPs can have positive effects on human health and are implicated in the reduction/prevention of chronic diseases, for which these molecules have gained importance as functional foods and nutraceuticals [20]. The different roles/activities attributed to the BPs are summarized in Figure 1; for instance, dairy products contain peptides exerting pharmacological effects similar to opium, also known as opioid peptides [21].

Moreover, antihypertensive peptides, useful in the treatment of hypertension disease due to the ability to inhibit the Angiotensin-I-Converting Enzyme (ACE), have been discovered in food proteins/peptides and are known as ACE inhibitory peptides [22,23]. Moreover, several ACE inhibitory peptides able to prevent or reduce hypertension have been isolated or extracted in water from different basidiomycetes’ edible mushroom fruiting bodies [9]. Several authors describe antifungal peptides, which act in different ways, such as nucleic acids synthesis inhibition or binding, protein synthesis blocking, membrane permeabilization, enzyme activity inhibition and apoptosis triggering [24,25,26]. Among the most studied peptides, there are antibiotics and antimicrobial peptides (AMPs). They are isolated from vertebrates (e.g., rabbit and human being part of immune defense system) and invertebrates (e.g., insects), as well as from plants, bacteria and fungi [27,28]. Apart from the antimicrobial activity, AMPs (natural or synthetic) are cytotoxic against tumor cell lines [29]. Indeed, peptides known as anticancer peptides (ACPs) are used in tumor treatment as adjuvant agents or potential drugs alternative to classical therapies [30], despite their susceptibility to proteolysis and poor cell membrane permeability [31], and sometimes also as immunoconjugates or while linked to chemotherapeutic agents [32]. Moreover, thrombotic disorders, including deep venous thrombosis and pulmonary embolism, can be treated by antithrombotic peptides derived from snake venom, centipedes and hookworms [33]. On the other hand, it is well-known that many peptides are natural antioxidants with health benefits due to their features (e.g., safety, low Mr and functional characteristics) and were found from different sources, such as plants (e.g., soybean, rapeseed and wheat [34]) as well as fungi [35]. Finally, the mineral-chelating property possessed by peptides is noteworthy, and several mineral-binding peptides have been produced following their isolation from different sources (e.g., milk, soybean, sea cucumber and eggs) [36,37,38,39]. Indeed, due to the presence of cysteinyl, histidinyl, serinyl, aspartatyl and glutamyl residues, peptides are able to chelate divalent metals, such as zinc and calcium ions, and can be used to enhance the bioavailability of minerals in human nutrition depending on the structural and functional relationship of metal-chelating peptides and the peptide–metal complexes’ stability in relation to gastrointestinal digestion [40].

## 4. Bioactive Peptides from Mushrooms

### 4.1. ACE Inhibitory Peptide from Agaricus bisporus

**Figure 2:**
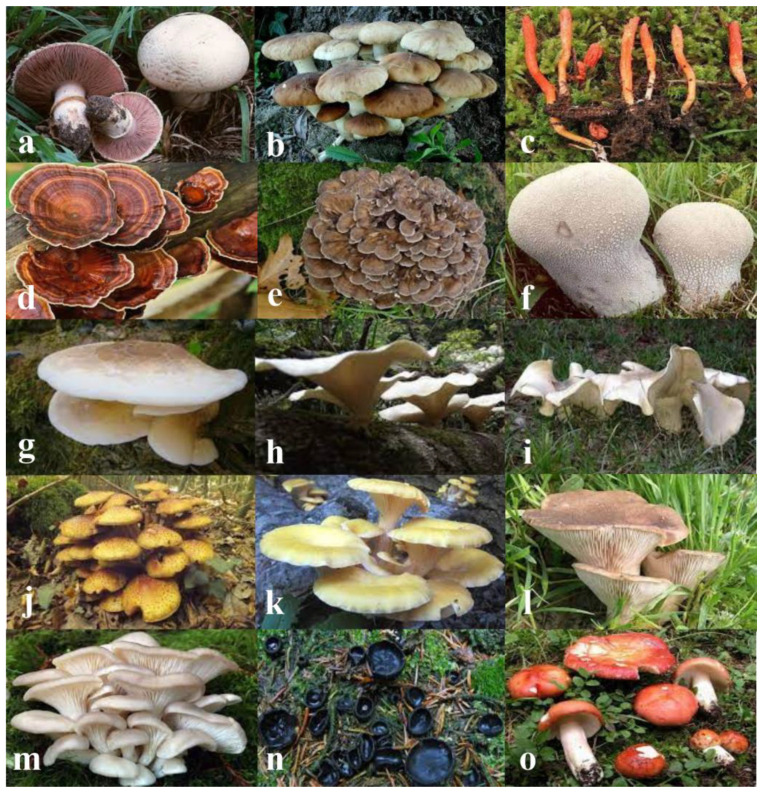
Photos of mushrooms that are sources of BPs described in the present review: (**a**) *Agaricus bisporus*; (**b**) *Cyclocybe aegerita*; (**c**) *Cordyceps militaris*; (**d**) *Ganoderma lucidum*; (**e**) *Grifola frondosa*; (**f**) *Handkea utriformis*; (**g**) *Hypsizygus marmoreus*; (**h**) *Lentinus sajor-caju*; (**i**) *Macrocybe gigantea*; (**j**) *Pholiota adiposa*; (**k**) *Pleurotus cornucopiae*; (**l**) *Pleurotus eryngii*; (**m**) *Pleurotus ostreatus*; (**n**) *Pseudoplectania nigrella* and (**o**) *Russula paludosa*.

*Agaricus bisporus* (J.E. Lange) Imbach 1946 is an edible basidiomycete mushroom (Table 1 and Figure 2). This mushroom grows in fertilized fields, dunghills, gardens and in meadows at the edge of the woods.

In 2014, Lau et al. isolated eight ACE inhibitory peptides from *A. bisporus*, selecting three more active peptides [41].

The molecular masses determined by mass spectrometry are equal to 605.30, 679.53 and 532.30 Da for peptide 1, 2 and 3, respectively, and the complete amino acid sequences achieved by LC-MS/MS are reported in Table 2. By using Lineweaver–Burk plots, the authors determined the ACE inhibitory mechanism, demonstrating that it was competitive for peptides 1 and 2 and non-competitive for peptide 3, with IC_50_ values of 63, 116 and 129 µM, respectively. All tested peptides showed high ACE inhibitory activity after in vitro gastrointestinal digestion [41].

### 4.2. Ubiquitin-Like Peptide and Agrocybin from Cyclocybe aegerita

*Cyclocybe aegerita* (V. Brig.) Vizzini 2014 is an edible basidiomycete mushroom (Table 1 and Figure 2) cultivated on agricultural and forest waste substrates, which are low-cost materials. The name of this mushroom has many synonyms, such as *Pholiota aegerita* (V. Brig.) Quél. 1872, *Agrocybe aegerita* (V. Brig.) Singer 1951 and *Agrocybe cylindracea* (DC.) Maire 1937 [42,43]. This mushroom grows on rotting trunks (but also trunks that are alive until the definitive attack of the fungus) of many broad-leaved trees, especially poplar, elm, elder and willow, in mild and humid weather, from spring to late autumn [44]. In the last few years, *C. aegerita* was extensively studied for the presence of enzymes known as ribotoxin-like proteins with a molecular weight of about 15 kDa, endowed with several biological actions (e.g., antiviral, antifungal and cytotoxic activities) and able to inhibit proteins synthesis [45,46,47]. Moreover, this mushroom, known as Pioppino in Italy, is considered a functional food rich in nutrients, such as amino acids, malic acid and sugars, with free radical scavenging power [48].

In 2003, Ngai et al. reported the presence of an ubiquitin-like peptide from the edible mushroom *C. aegerita* [49]. By gel filtration and SDS-PAGE analysis, this peptide shows a molecular mass of about 9500 Da, while the N-terminal amino acid sequence obtained by automated Edman degradation, reported in Table 2, seems to be identical to ubiquitin from several organisms. This peptide has a good thermal stability up to 60 °C, has an optimal pH equal to 6.0 and exhibits ribonucleolytic activity toward the various polyhomoribonucleotides, with higher activity on the polyC (84.4 U/mg), followed by polyU, polyA and polyG (15, 5.3 and 1.8 U/mg, respectively). Finally, the ubiquitin-like peptide from *C. aegerita* possesses an inhibitory effect on the proliferation of M-1 myeloid leukemia and HepG2 cell lines, with an IC_50_ value of 10 and 100 µM, respectively [49].

In 2005, Ngai et al. reported the presence of a BP named agrocybin in the edible mushroom *C. aegerita* [50]. This peptide shows a molecular mass of about 9000 Da as determined by SDS-PAGE analysis and gel filtration, while the N-terminal amino acid sequence obtained by automated Edman degradation is reported in Table 2. Agrocybin possesses antifungal activity against *Mycosphaerella arachidis*, a fungal plant pathogen, with an IC_50_ value of 125 µM. This activity was tested at different temperatures (0, 20, 40, 60, 80 and 100 °C), and agrocybin is able to inhibit the mycelia grow up to 80 °C, while no inhibiting effect is shown after treating the peptide at a temperature of 100 °C. Moreover, agrocybin inhibits HIV-1 reverse transcriptase with an IC_50_ of 60 µM. On the other hand, this toxin tested at a concentration up to 300 µM did not exert any activity toward several bacteria strains, both Gram-positive (*Bacillus cereus, Bacillus megaterium, Bacillus subtilis, Mycobacterium phlei* and *Staphylococcus aureus*) and Gram-negative (*Enterobacter aerogenes, Escherichia coli, Proteus vulgaris, Pseudomonas aeruginosa* and *Pseudomonas fluorescens*). Finally, agrocybin did not possess antiproliferative activity towards Hep G2 cell lines when tested up to 110 µM [50].

### 4.3. Cordymin from Cordyceps militaris

*Cordyceps militaris* (L.) Fr. 1818 is an ascomycete edible mushroom originally described by Carl Linnaeus in 1753 as *Clavaria militaris* (Table 1 and Figure 2) [51,52]. This mushroom is found in woods, meadows and gardens in summer and autumn, and the mycelium is pathogenic for insects. *C. militaris* is widely used in traditional Chinese medicine and as a crude drug and popular tonic food in East Asia [53].

In 2011, Wong et al. isolated a biological peptide named cordymin from *C. militaris* [54]. The molecular mass of this peptide, corresponding to 10,906 Da as determined by MALDI-TOF mass spectrometry, is close to the limit for considering this peptide as a protein. The N-terminal amino acid sequence of cordymin achieved by automated Edman degradation is reported in Table 2. This peptide is able to inhibit the mycelia growth of numerous pathogen fungal species with IC_50_ values ranging from 10 to 750 µM (see Table 3), has stable activity across a range of pH values and is heat-resistant and not metal-dependent [54]. In addition, cordymin is able to inhibit HIV-1 reverse transcriptase with an IC_50_ value of 55 µM and reduce the proliferation of MCF-7 breast cancer cells but not of HT-29 colon cancer cells. Finally, cordymin does not present protease activity toward casein [54].

### 4.4. GLP fraction from Ganoderma lucidum

*Ganoderma lucidum* (Curtis) P. Karst. 1881 is an edible basidiomycete and saprophytic fungus (Table 1 and Figure 2) that grows in late spring, summer and autumn on broad-leaved trees, especially oak and chestnut, and sometimes even olive trees. *G. lucidum* is widely used in oriental medicine due to its numerous pharmacological effects, e.g., antitumor and antiviral activities, as well as its potential immunomodulating and antihypertension properties. The mushroom is highly studied for the presence of several natural bioactive components, mostly polysaccharides and triterpenoids used as supplemental therapies in several diseases [55].

In 2004, Sun et al. isolated a BP fraction named GLP (*G. lucidum* peptide) from *G. lucidum* [35]. The peptides fraction has a molecular mass <10,000 Da, while the N-terminal sequence is unknown (Table 2). GLP fraction displays high antioxidant activity comparable to that of the synthetic antioxidant butylhydroxytoluene (BHT) in both soybean oil and lard systems. In particular, the authors show that GLP fraction at 0.1% (w:w) after 13 days inhibited the oxidation of soybean oil and lard at a rate of 60 and 30%, respectively, likely because soybean is rich in polyunsaturated fatty acids. GLP fraction blocked the oxidation of polyunsaturated fatty acids responsible for lipid hydroperoxides formation by inhibiting 90% of lipoxygenase (LOX) activity in vitro at 0.3 mg/mL. Moreover, using mouse liver homogenates, 0.7 mg/mL of GLP fraction inhibited 72% of malondialdehyde (MDA) formation induced by H_2_O_2_ resulting from lipid peroxidation of polyunsaturated fatty acids. Finally, although the antioxidant properties of *G. lucidum* are mainly attributed to the water-soluble polysaccharides fraction, the authors demonstrated that the low-molecular-weight water extracts, mostly constituted of peptides, displayed greater antioxidant activity with respect to the high-molecular-weight fractions [35].

### 4.5. ACE Inhibitory Peptide from Grifola frondosa

*Grifola frondosa* (Dicks.) Gray 1821 is an edible basidiomycete mushroom typically found from late summer to early autumn (Table 1 and Figure 2). It is native to China, Europe and North America.

In 2001, Choi et al. isolated an ACE inhibitory peptide from *G. frondosa* [56]. The theoretical molecular mass of the peptide corresponding to 567.30 Da [M+H^+^]^+^ was achieved with the Expasy PeptideMass software (https://web.expasy.org/peptide_mass/; accessed on 7 September 2021), and the complete amino acid sequence achieved by Edman degradation is reported in Table 2. The authors determined the competitive ACE inhibitory pattern using Lineweaver–Burk plots. Moreover, the inhibitory activity has an IC_50_ value of 0.13 mg and was also maintained after exposing the peptide to proteolytic enzymes [56].

### 4.6. CULP from Handkea utriformis

*Handkea utriformis* (Bull.) Kreisel 1989 is an edible basidiomycete mushroom (Table 1 and Figure 2). The name of this mushroom has many synonyms, such as *Calvatia utriformis* (Bull.: Pers.) Jaap 1918, *Calvatia caelata* (Bull.) Morgan 1890 and *Lycoperdon utriforme* (Bull.) 1790. This mushroom is very common, both solitary and gregarious, and it grows in meadows and pastures from spring to autumn [57].

*H. utriformis* has globose fruiting bodies that are white and edible when young, while during maturation, it produces dark and powdery spores due to the autolysis process. Besides its use for culinary purposes, *H. utriformis* is highly employed in traditional Chinese medicine to treat several diseases such as coughing, stomach ache and fever [57,58].

**Figure 3:**
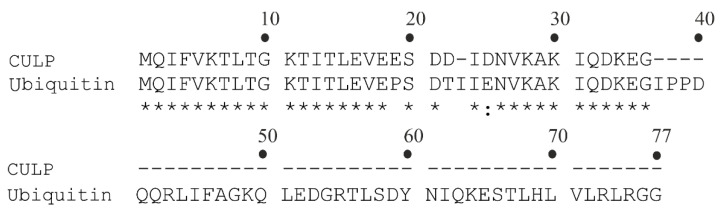
CULP and ubiquitin amino acid sequence alignment [59]. The standard one-letter code was used for the amino acid residues; identical residues (*) and conserved substitutions (:) are reported.

In 2001, Lam et al. isolated a BP from *H. utriformis* [59]. The molecular mass equal to 8500 Da was determined by SDS-PAGE analysis. Moreover, the N-terminal sequence of the peptide, achieved by automated Edman degradation and reported in Table 2, shares 32 out of 35 amino acid residues with the N-terminal of ubiquitin, as highlighted in Figure 3. In light of this ~91.4% identity, the peptide was named ubiquitin-like peptide isolated from *C. caelata* (synonym of *H. utriformis*), abbreviated as CULP. It displays N-glycosylase activity, inhibits protein synthesis in a rabbit reticulocyte lysate cell-free system (~38% at 14 µM) and possesses ribonuclease activity of 1 IU/mg toward yeast tRNA. Moreover, CULP exerted antiproliferative and antimitogenic activities against human breast carcinoma cells and murine splenocytes, respectively (IC_50_ = 0.1 µM for both activities) [59].

### 4.7. ACE Inhibitory Peptide from Hypsizygus marmoreus

*Hypsizygus marmoreus* (Peck) H.E. Bigelow 1976 is an edible mushroom found in Korea, Japan, China, Northern Europe and East Asia (Table 1 and Figure 2). It grows well in beech stumps, withered maple and trees [60].

In 2013, Kang et al. isolated an ACE inhibitory peptide from *H. marmoreus* [61]. The complete amino acid sequence of this peptide is reported in Table 2. The molecular mass as determined by LC-MS/MS was reported to be equal to 567.30 Da even if, when we performed the analysis of the amino acid sequence with the Expasy PeptideMass software, the theoretical molecular mass of the peptide was 949.09 Da [M+H^+^]^+^ (https://web.expasy.org/peptide_mass/; accessed on 7 September 2021).

Following the peptide chemical synthesis, the authors determined the ACE inhibitory activity with an IC_50_ value of 0.19 mg/mL, and *H. marmoreus* H_2_O extract is able to inhibit hypertension in spontaneously hypertensive rats [61].

### 4.8. PSULP from the Mushroom Lentinus sajor-caju

*Lentinus sajor-caju* (Fr.) Fr. 1838, synonym *Pleurotus sajor-caju*, is an edible basidiomycete mushroom (Table 1 and Figure 2).

In 2002, Ng et al. isolated an ubiquitin-like peptide from *L. sajor-caju* [62]. This peptide, named *P. sajor-caju* ubiquitin-like peptide (PSULP), shows a molecular mass of about 9500 Da, determined by SDS-PAGE analysis and gel filtration, while the N-terminal amino acid sequence achieved by automated Edman degradation is identical to that of ubiquitin and is reported in Table 2. PSULP is able to inhibit translation in a cell-free reticulocyte lysate system with an IC_50_ value of 30 nM and exhibits ribonucleolytic activity of 450 IU/mg when assayed against tRNA from yeast [62].

### 4.9. ACE Inhibitory Peptide from Macrocybe gigantea

*Macrocybe gigantea* (Massee) Pegler & Lodge 1998, synonym *Tricholoma giganteum*, is an edible basidiomycete mushroom native to India, Pakistan and Nepal (Table 1 and Figure 2). This mushroom grows in groups or sometimes fairy rings in shady or grassy areas, or in association with angiosperm trees.

In 2004, Lee et al. isolated an ACE inhibitory peptide from *M. gigantea* [63]. The molecular mass was determined by LC-MS is equal to 301.00 Da, and the complete amino acid sequence of the tripeptide achieved by automated Edman degradation is reported in Table 2. The authors determined the competitive ACE inhibitory pattern using Lineweaver–Burk plots. Moreover, the inhibitory activity has an IC_50_ value of 0.31 mg and was also maintained after subjecting the peptide to proteolytic enzymes. Finally, the peptide inhibits hypertension in spontaneously hypertensive rats [63].

### 4.10. ACE Inhibitory Peptide from Pholiota adiposa

*Pholiota adiposa* (Batsch) P.Kumm. 1871 is an inedible basidiomycete mushroom that grows in temperate climate regions, having an important role in the ecosystem as a wood decomposer and soil saprotroph (Table 1 and Figure 2).

In 2006, Kyo-Chul et al. isolated an ACE inhibitory peptide from *Pholiota adiposa* [64]. The molecular mass as determined by MALDI-MS is equal to 414.00 Da, and the complete amino acid sequence of the pentapeptide achieved by Edman degradation is reported in Table 2. The authors determined the competitive ACE inhibitory pattern using Lineweaver–Burk plots, and the inhibitory activity has an IC_50_ value of 0.04 mg; the peptide inhibits hypertension in spontaneously hypertensive rats (dosage 1 mg kg^−1^) [64].

### 4.11. ACE Inhibitory Peptide from Pleurotus cornucopiae

*Pleurotus cornucopiae* (Paulet) Rolland 1910 is an edible basidiomycete mushroom that grows bushy on broad-leaved stumps from spring to autumn (Table 1 and Figure 2).

In 2011, Jang et al. isolated two ACE inhibitory peptides from *Pleurotus cornucopiae* [65]. The molecular masses of ACE inhibitory peptides 1 and 2, determined by MS/MS, are equal to 1622.85 and 2037.26 Da, respectively, and the complete amino acid sequences of the peptides, achieved by MS/MS on a LCQ Deca ESI ion trap mass spectrometer, are reported in Table 2. The authors determined the competitive ACE inhibitory pattern using Lineweaver–Burk plots, and the inhibitory activity of ACE inhibitory peptides 1 and 2 has IC_50_ values of 0.45 and 1.10 mg, respectively. Finally, the water extract from *P. cornucopiae* inhibits hypertension in spontaneously hypertensive rats (dosage 600 mg kg^−1^) [65].

### 4.12. Eryngin from Pleurotus eryngii

*Pleurotus eryngii* (DC.: Fr.) Quél. 1872 is an edible basidiomycete mushroom typical of the Mediterranean region as well as Central Europe, Central Asia and North Africa (Table 1 and Figure 2) [66]. The fungus, rarely attacked by larvae, is widespread in the regions of Southern Italy, where it is appreciated for its flavor and aroma [67]. It grows from spring to autumn in uncultivated fields and pastures, and it behaves from an ecological point of view most like a saprophytic–parasitic fungus, in association and often with growth on the roots of the perennial plant *Eryngium campestre* L. (known as field eryngo) [66]. This mushroom is studied for the presence of eryngeolysin, an antibacterial and hemolytic monomeric protein with a molecular weight of 17 kDa. This protein displays a similarity to the N-terminal amino acid sequences of ostreolysin from *Pleurotus ostreatus* and agerolysin from *C. aegerita* [68].

In 2004, Wang et al. isolated a BP named eryngin from *P. eryngii* [69]. The molecular mass, determined by gel filtration and SDS-PAGE analysis, is equal to 10,000 Da. Moreover, the N-terminal sequence achieved by automated Edman degradation is reported in Table 2. Eryngin possesses antifungal activity against *Fusarium oxysporum* and *Mycosphaerella arachidicola* with IC_50_ values of 1.35 and 3.5 µM, respectively [69]. Moreover, eryngin did not possess lectin or ribonuclease activities when tested at both 10 and 100 µM concentrations [69].

### 4.13. POP and Pleurostrin from Pleurotus ostreatus

*Pleurotus ostreatus* (Jacquin: Fr.) Kummer 1871 is an edible basidiomycete mushroom (Table 1 and Figure 2) [70]. It grows throughout the winter on dead poplar, willow, beech, oak, maple, birch and mulberry wood, and sometimes even on living trunks [70]. It can be found isolated or, more frequently, in groups, even growing bushy.

In 2002, Ye and Ng isolated a BP named POP (*P. ostreatus* peptide) from *P. ostreatus* [71]. This peptide shows a molecular mass of about 9000 Da, as determined by SDS-PAGE analysis and gel filtration; its N-terminal amino acid sequence obtained by automated Edman degradation is reported in Table 2. POP possessed a 651 U/mg RNase activity toward yeast transfer RNA and caused a dose-dependent inhibition of protein synthesis in rabbit cell-free reticulocyte lysate systems with an IC_50_ value of 15 nM [71].

In 2005, Chu et al. isolated a BP named pleurostrin from *P. ostreatus* [72]. The molecular mass equal to 7000 Da was determined by SDS-PAGE and gel filtration using a FPLC by Superdex 75 column, while the N-terminal amino acid sequence achieved/obtained by automated Edman degradation is reported in Table 2. Pleurostrin exhibits antifungal activity against *M. arachidicola, F. oxysporum* and *Physalospora piricola* with percentages of growth inhibition of 45, 20 and 63%, respectively. Moreover, pleurostrin is able to inhibit protein synthesis in a cell-free reticulocyte lysate system with an IC_50_ value of 28 µM, and it exhibits anti-proliferative activity on MBL2 and L1210 leukemia cells with IC_50_ values of 15 and 41 µM, respectively [72]. Finally, it is worth mentioning that several toxic proteins have been isolated from *P. ostreatus*, such as the ribotoxin-like protein ostreatin [73] and two pore-forming proteins named ostreolysin [74] and pleurotolysin [75].

### 4.14. Plectasin from Pseudoplectania nigrella

*Pseudoplectania nigrella* (Pers.) Fuckel 1870 is a black saprophytic inedible ascomycete fungus found on the floor of northern European pine forests (Table 1 and Figure 2). This fungus is distributed worldwide (North America, the Caribbean, Europe, India, Madagascar, New Zealand and Japan) [76].

In 2005, Mygind et al. identified a BP named plectasin from *P. nigrella* mRNA [77]. The molecular mass, determined by LC-ESI-QTOF-MS/MS, is equal to 4398.80 Da. Moreover, N-terminal sequencing was done on a Procise automatic sequencer (Applied Biosystems), while the complete putative amino acid sequence of the mature peptide, obtained by cDNA sequencing, is reported in Table 2.

Plectasin is considered a bactericide, exerting antimicrobial activity mainly against a plethora of tested Gram-positive bacteria at physiological ionic strength, conversely from most vertebrate defensins, which need very low ionic strength. On the other hand, this peptide is selective for bacteria cells with respect to mammalian cells in vitro, since it did not display cytotoxicity against murine L929 fibroblasts or normal human epidermal keratinocytes, as well as not exerting hemolytic action toward human erythrocytes. Finally, an in vivo study using mouse models of pneumococcal peritoneal infection highlighted the anti-infective action of plectasin [77].

Due to its therapeutic potential, plectasin is considered a novel antimicrobial peptide with excellent penetration into cerebrospinal fluid, suggesting its potential effectiveness in the treatment of CNS infections caused by Gram-positive pathogens, such as pneumococcal meningitis [78].

### 4.15. SU2 peptide from Russula paludosa

*Russula paludosa* Britzelm. 1891 is a wild edible basidiomycete mushroom (Table 1 and Figure 2) [79]. This mushroom is a mycorrhizal fungus that grows in coniferous woods (preferably under Pinus spp.) near blueberry or in sphagnum trees, in peat bogs and in marshes or marshy areas in Europe, North America and other similar habitats. It bears fruit in the period between summer and autumn, and in certain areas, it can be a very common fungus.

In 2007, Wang et al. isolated SU2 peptide from *R. paludosa* [80]. This peptide has a molecular mass estimated by SDS-PAGE analysis and gel filtration to be about 4500 Da, while the N-terminal amino acid sequence achieved by automated Edman degradation is reported in Table 2. SU2 exhibited HIV-1 reverse transcriptase inhibitory activity with a IC_50_ of 11 mM [80]. Moreover, the authors performed several assays in order to test hem-agglutinant, laccase, ribonuclease, antifungal, protease and protease inhibitory activities; however, the peptide did not show any of these activities.

## 5. Major Findings

As shown in Table 2, the revisiting of literature allowed us to identify 20 different BPs from 15 different fungal species, highlighting that mushrooms represent a rich source of BPs with several biological activities. A comparison of different fungal sources revealed that two members belong to phylum Ascomycota (Hypocreales and Pezizales orders) and thirteen to phylum Basidiomycota, among which the most representative orders are Agaricales (nine members) followed by Polyporales (three members) and Russulales (one member). Apart from the inedible *P. adiposa* and *P. nigrella*, the other mushroom sources of BPs are edible. Considering the different biological activities, from our search it has emerged that 45% of BPs possessed ACE inhibitory activity, while about 35% showed antifungal, antiviral and anticancer activities, and the others displayed antioxidant or antibiotic and antimicrobial properties (GLP fraction and plectasin, respectively). Amino acid composition and the chain length and sequence of ACE inhibitory peptides are considered the most important structural characteristics responsible for their inhibitory potential, although the structural/functional relationship for these peptides has not yet been completely elucidated [81]. In this framework, ACE inhibitory peptides from mushrooms are short-chain peptides with 3 to 17 amino acid residues, in agreement with previous crystallography studies, in which it was demonstrated that large peptides are unable to bind to ACE active sites [82]. Most of them contain at their C-terminus a lysine or proline, which have been indicated as the most effective amino acid residues in increasing ACE inhibitory effects [83]. The inhibition pattern of ACE inhibitory peptides can be determined spectrophotometrically by measuring the absorbance of hippuric acid liberated at 228 nm after incubation of peptides with ACE solution, using HHL as substrate [84]. As determined by Lineweaver–Burk plots, the ACE inhibition can be: (i) competitive when the inhibitor binds competitively with the substrate at the level of the ACE active site or (ii) non-competitive when the inhibitor interacts with both the free enzyme and the enzyme–substrate complex. The IC_50_ values representing the concentration of the ACE inhibitor required to inhibit 50% of ACE activity, reported in Table 4, showed that the tripeptide Gly-Gln-Pro from *M. gigantea* has the most potent inhibitory activity, while the Arg-Leu-Ser-Gly-Gln-Thr-Ile-Glu-Val-Thr-Ser-Glu-Tyr-Leu-Phe-Arg-His peptide from *P. cornucopiae* has the least potent.

Moreover, the complete amino acid sequences of all the ACE inhibitory peptides allowed us to calculate their theoretical isoelectric point (pI) and the grand average of hydropathicity (GRAVY) index (Table 4). The molecular weights of the reviewed ACE inhibitory peptides ranged from 301 to 2037.26 Da (Table 2). Among them, the molecular weights of pentapeptides and hexapeptides, which were the most abundant, ranged from 414.42 to 747.89 Da (Table 2). On the other hand, the pI of the reviewed ACE inhibitory peptides ranged from 5.52 to 9.75, while the hydrophobicity values ranged from −2.12 to 1.24, suggesting a prevalence of hydrophilic nature for these peptides, according to what was previously reported [85].

Overall, mushroom-derived ACE inhibitory peptides are resistant to intestinal proteases digestion and have no adverse side effects (e.g., allergic reactions), indicating a potential as ingredients in functional foods or as effective potential antihypertensive drugs.

The structural analysis of the BP plectasin isolated from *P. nigrella* revealed that this peptide consists of an α/β motif with an α-helix and two antiparallel β-strands, while the sequence, similar to invertebrate defensins, is characterized by six cysteinyl residues, forming 3 disulfide bridges that stabilize the structure [77]. Moreover, plectasin is weakly basic (pI = 7.77), and the calculated negative GRAVY index of −0.695 suggests an overall hydrophilic nature for this peptide.

Finally, the lack of complete information on amino acid sequences for the remaining reviewed BPs did not allow us to properly analyze and discuss their physico-chemical properties. However, considering the data reported, we hypothesize that most of peptides obtained have a positive net charge and are rich in basic amino acids. Overall, the presence of basic amino acids could justify the interaction with negative charges of the membrane phospholipids in order to better execute their biological action [86].

## 6. Isolation and Purification of Fungal Peptides

Although peptides consist of amino acids linked by covalent bonds, their structure depends on the presence of additional weak linkages such as hydrogen, hydrophobic or electrovalent bonds. Since the biological activity of a peptide depends on the integrity of its molecular conformation, the methods for their isolation must be sufficiently mild so as not to disrupt these weak bonds and cause denaturation. The determination of the optimum conditions for peptide extraction is semi-empirical and may be extremely laborious. In general, peptides are sensitive to heat, extremes of pH and the presence of high concentrations of organic solvents or detergents. Before extracting peptides from fungal tissues, the cellular structure must be destroyed; in most of cases, the tissue may be macerated in the presence of an aqueous diluent (usually at a ratio of 1:3; *w*:*v*) in a blender fitted with high-speed rotating knives.

The main methods used for peptide separation are based on the peptides’ physico-chemical properties, e.g., charge that could be modified by pH changing, polarity, molecular size, solubility and specific covalent or non-covalent interactions. In light of these characteristics, different chromatographic applications were used to obtain high-purity peptides, such as RP-HPLC chromatography, hydrophobic interaction chromatography and affinity chromatography, based on specific interaction, as well as gel filtration and ion-exchange chromatography, based on molecular size or charge, respectively [87].

**Figure 4:**
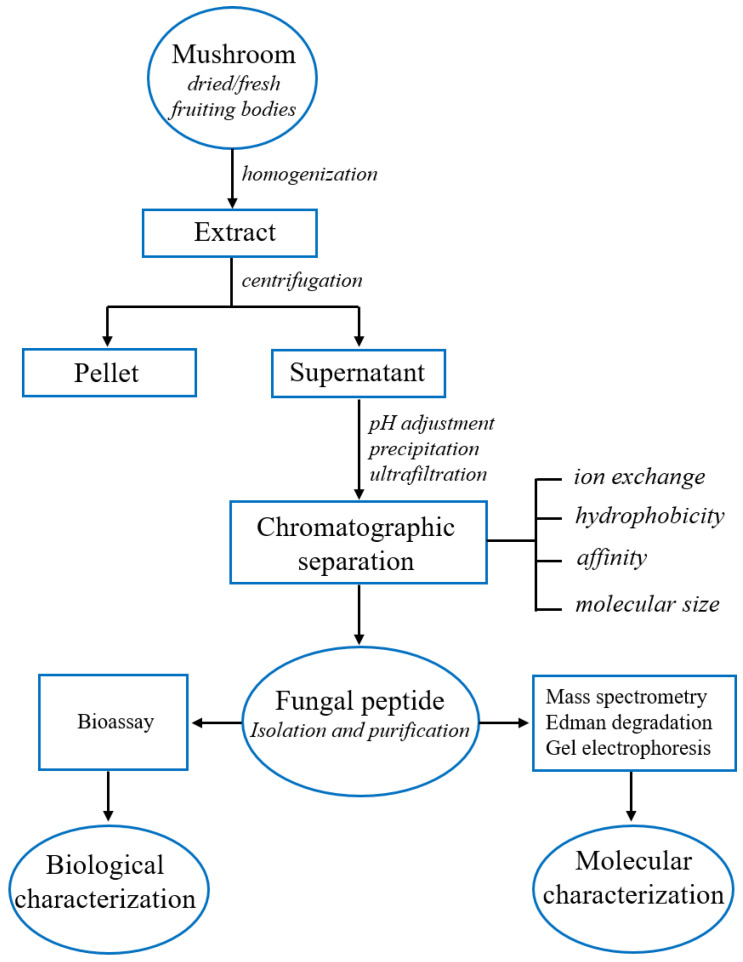
Flowchart for peptide extraction, isolation, purification and molecular characterization.

Considering the most relevant literature, the general basic principles for peptide extraction, isolation, purification and characterization have been summarized in Figure 4. The first step of the process consists of the disruption of the cells containing the peptides, generally by using a Waring blender or a mortar and pestle chilled with liquid nitrogen and an appropriate lysis buffer. Finally, the cell debris can be removed by centrifugation so that the peptides and other soluble compounds remain in the supernatant. It is recommended to add protease inhibitor cocktails or proceed quickly, keeping the extract cooled, to avoid peptide digestion. Optionally, the extract containing the peptides is concentrated by ultrafiltration using a 1000 Da membrane. A common step to isolate peptides is precipitation using a salt such as ammonium sulfate (NH_4_)_2_SO_4_, performed by adding increasing amounts of ammonium sulfate up to 100% of salt saturation and collecting the different fractions of precipitate peptides. Ammonium sulfate is commonly used due to the fact that is has a high solubility in water, is not harmful to most proteins/peptides and can be removed by dialysis. Followed precipitation, the peptides are recovered by centrifuging at 4 °C for 15 min at 10,000× *g* and dialyzed against an appropriate buffer. Generally, four different properties can be used to separate peptides. Firstly, peptides may be purified according to their pI by ion-exchange column, in which the compounds are separated according to the nature and degree of their ionic charge. Secondly, peptides can be separated according to their size or molecular weight by size exclusion chromatography (SEC), also known as gel filtration, in which the smaller molecules having to traverse a larger volume in a porous matrix elute and are later separated based on their size. Thirdly, peptides may be separated by polarity/hydrophobicity by high-performance liquid chromatography (HPLC) or reversed-phase HPLC (RP-HPLC). Finally, the affinity can be exploited to make a specific and reversible binding of a peptide to a matrix-bound ligand in order to isolate the molecule of interest by affinity chromatography. One or more chromatographic steps can be combined to obtain a higher quality of separation. Peptides’ molecular characterization can be obtained by several techniques. Sodium dodecyl sulfate–polyacrylamide gel electrophoresis (SDS-PAGE), gel filtration and MALDI-MS have been used for molecular weight determination, while Edman degradation and LC-MS/MS are the most important methodologies to achieve the complete amino acid sequence. Finally, the biological activity of each purified peptide, such as antihypertensive, antifungal, antibiotic and antimicrobial, anticancer, antiviral, antioxidant and ACE inhibitory properties can be attributed by using an appropriate enzymatic assay [88].

As an example, Ngai et al. report that for the obtainment of agrocybin, *C. aegerita* fresh fruiting bodies were homogenized in deionized water in a ratio of 1:3 (*w*:*v*) using a Waring blender and subsequently centrifuged at 4 °C for 30 min at 12,000× *g*. The supernatant was adjusted up to pH 7.4 at a final concentration of 20 mM using Tris•Cl buffer and subjected to anion exchange chromatography by DEAE-cellulose, washing off the unabsorbed material with 20 mM Tris•Cl, pH 7.4. This fraction was further subjected to Affi-gel blue gel affinity chromatography, eluted with 20 mM Tris•Cl, pH 7.4 containing 1 M NaCl, dialyzed and applied to a cation-exchange chromatography Mono S column, eluted with a linear gradient of NaCl (0–0.3 M). The major peak was further dialyzed and gel-filtered on a Superdex-75 column in 20 mM NH_4_HCO_3_ buffer at pH 9.4 to obtain the purified agrocybin peptide [50]. A similar procedure, sometimes with a few modifications, has also been applied for the obtainment of: (i) ubiquitin-like peptides from *C. aegerita* and *L. sajor-caju* fresh fruiting bodies [49,62]; (ii) CULP from *H. utriformis* fresh fruiting bodies [59]; (iii) eryngin from *P. eryngii* fresh fruiting bodies [69]; (iv) pleurostrin from *P. ostreatus* fresh fruiting bodies [72] and (v) SU2 peptide from *R. paludosa* dried fruiting bodies [80]. On the other hand, the protocols for ACE inhibitory peptide extraction usually start from dried materials. Briefly, dried fruiting bodies are homogenized in water in a ratio of 1:40 (*w*:*v*), stirred 12 h at 30 °C, centrifuged 20 min at 10,000× *g* and subjected to ultrafiltration using a 5000 Da membrane. The filtrate is subjected to gel filtration (e.g.: Sephadex G-25) and one to three steps of RP-HPLC using a C18 column equilibrated in H_2_O/TFA 0.1% and eluted with a linear gradient (0–100% in CH_3_CN containing TFA 0.1%) [41,56,61,63,64,65]. Finally, plectasin from *P. nigrella* can be obtained in recombinant form from an *Aspergillus oryzae* expression system [77].

## 7. Concluding Remarks

The present work aims to review all the BPs isolated and characterized from mushrooms in the last 20 years, focusing our analysis on amino acid polymers with a molecular weight up to 10 kDa, according to the accepted biochemical definition of ‘peptide’.

The biological action of these peptides has a broad spectrum of functions, including antihypertensive, antifungal, antibiotic and antimicrobial, anticancer, antiviral, antioxidant and ACE inhibitory properties. Overall, their possible biotechnological/medical applications should stimulate even more the search of novel BPs yet to be discovered, considering that fungal organisms are a rich reservoir of these molecules.

At the same time, we hope that the present review encourages researchers to correctly use the term ‘peptide’ as accepted by the biochemical community, as it is also often improperly adopted for proteins with molecular weight higher than 10 kDa.

## Figures and Tables

**Table 1:** Taxonomic classification of mushrooms that are sources of BPs belonging to domain Eukaryota and kingdom Fungi.

Scientific Name	Common Name	Phylum	Class	Order	Family
*Agaricus bisporus*	button mushroom	Basidiomycota	Agaricomycetes	Agaricales	Agaricaceae
*Cyclocybe aegerita*	poplar mushroom	Basidiomycota	Agaricomycetes	Agaricales	Strophariaceae
*Cordyceps militaris*	caterpillar fungus	Ascomycota	Ascomycetes	Hypocreales	Clavicipataceae
*Ganoderma lucidum*	lingzhi	Basidiomycota	Agaricomycetes	Polyporales	Ganodermataceae
*Grifola frondosa*	hen-of-the-woods	Basidiomycota	Agaricomycetes	Polyporales	Grifolaceae
*Handkea utriformis*	mosaic puffball	Basidiomycota	Agaricomycetes	Agaricales	Lycoperdaceae
*Hypsizygus marmoreus*	beech mushroom	Basidiomycota	Agaricomycetes	Agaricales	Lyophyllaceae
*Lentinus sajor-caju*	white-rot fungus	Basidiomycota	Agaricomycetes	Polyporales	Polyporaceae
*Macrocybe gigantea*	giant mushroom	Basidiomycota	Agaricomycetes	Agaricales	Catathelasmataceae
*Pholiota adiposa*	chestnut mushroom	Basidiomycota	Agaricomycetes	Agaricales	Strophariaceae
*Pleurotus cornucopiae*	branched oyster mushroom	Basidiomycota	Agaricomycetes	Agaricales	Pleurotaceae
*Pleurotus eryngii*	king oyster mushroom	Basidiomycota	Agaricomycetes	Agaricales	Pleurotaceae
*Pleurotus ostreatus*	oyster mushroom	Basidiomycota	Agaricomycetes	Agaricales	Pleurotaceae
*Pseudoplectania nigrella*	ebony cup	Ascomycota	Pezizomycetes	Pezizales	Sarcosomataceae
*Russula paludosa*	hintapink	Basidiomycota	Basidiomycetes	Russulales	Russulaceae

**Table 2:** N-terminal amino acid sequence of BPs from mushrooms reported in literature. ‘NCBI: txid’ refers to the taxonomy ID of mushrooms reported in NCBI taxonomy browser. The distinctive anionic tetrapeptide (DEDD) motif of plectasin is highlighted.

Peptide Name	Peptide M*r* (Da)	N-Terminal Sequence	Peptide Source	Taxonomy NCBI: Txid	Ref.
ACE inhibitory peptide 1	605.30	_1_ RIGLF _5_ ^a^	*Agaricus bisporus*	5341	Lau et al., 2014
ACE inhibitory peptide 2	679.53	_1_ AHEPVK _6_ ^a^	*Agaricus bisporus*	5341	Lau et al., 2014
ACE inhibitory peptide 3	532.30	_1_ PSSNK _5_ ^a^	*Agaricus bisporus*	5341	Lau et al., 2014
Ubiquitin-like peptide	9500	_1_ MQIFVK _6_	*Cyclocybe aegerita*	5400	Ngai et al., 2003
Agrocybin	9000	_1_ ANDPQCLYGN VAAKF _15_	*Cyclocybe aegerita*	5400	Ngai et al., 2005
Cordymin	10,906	_1_ AMAPPYGYRT PDAAQ _15_	*Cordyceps militaris*	73501	Wong et al., 2011
GLP fraction	<10,000	n.r.	*Ganoderma lucidum*	5315	Sun et al., 2004
ACE inhibitory peptide	747.42 ^b^	_1_ VIEKYP _6_ ^a^	*Grifola frondosa*	5627	Choi et al., 2001
CULP	8500	_1_ MQIFVKTLTG KTITLEVEES DDIDNVKAKI QDKEG _35_	*Handkea utriformis*	258083	Lam et al., 2001
ACE inhibitory peptide	567.30 ^b^	_1_ LSMGSASLSP _10_ ^a^	*Hypsizygus marmoreus*	39966	Kang et al., 2013
PSULP	9500	_1_ MQIFVKTLTG KTITL _15_	*Lentinus* *sajor-caju*	50053	Ng et al., 2002
ACE inhibitory peptide	301.00	_1_ GQP _3_ ^a^	*Macrocybe gigantea*	1491104	Lee et al., 2004
ACE inhibitory peptide	414.00	_1_ GQGGP _5_ ^a^	*Pholiota adiposa*	64639	Koo et al., 2006
ACE inhibitory peptide 1	1622.85	_1_ RLPSEFDLSA FLRA _14_ ^a^	*Pleurotus cornucopiae*	5321	Jang et al., 2011
ACE inhibitory peptide 2	2037.26	_1_ RLSGQTIEVT SEYLFRH _17_ ^a^	*Pleurotus cornucopiae*	5321	Jang et al., 2011
Eryngin	10,000	_1_ ATRVVYCNRR SGSVVGGDDT VYYEG _25_	*Pleurotus eryngii*	5323	Wang et al., 2004
POP	9000	_1_ GPCYLVAFYE SSGRR _15_	*Pleurotus ostreatus*	5322	Ye et al., 2002
Pleurostrin	7000	_1_ VRPYLVAF _8_	*Pleurotus ostreatus*	5322	Chu et al., 2005
Plectasin	4398.80	_1_ GFGCNGPWDE DDMQCHNHCK SIKGYKGGYC AKGGFVCKCY _40_ ^a^	*Pseudoplectania nigrella*	96584	Mygind et al., 2005
SU2 peptide	4500	_1_ KREHGQHCEF _10_	*Russula paludosa*	176813	Wang et al., 2007

n.r., not reported. ^a^ Complete amino acid sequence. ^b^ Experimental molecular mass not in accordance with the theoretical molecular peptide mass 949.09 Da [M+H^+^]^+^. For further details, see paragraph 4.7.

**Table 3:** Half-maximal inhibitory concentration (IC_50_) derived from antifungal activity of cordymin tested against several pathogen fungal species.

Organism	Fungal Species	Diseases	IC_50_ (µM)	Ref.
Filamentous fungus	*Bipolaris maydis*	Plant pathogen causing Southern corn leaf blight (SCLB) and stalk rot diseases	50	Wong et al., 2011
Filamentous fungus	*Mycosphaerella arachidicola*	Plant pathogen causing plant disease	10	Wong et al., 2011
Filamentous fungus	*Rhizoctonia solani*	Plant pathogen causing collar rot, root rot, damping off and wire stem diseases	80	Wong et al., 2011
Unicellular fungus (planktonic form)	*Candida albicans*	Human pathogen causing opportunistic infections	750	Wong et al., 2011

**Table 4:** Structural characteristics and inhibition of ACE inhibitory peptides from mushrooms.

ACE Inhibitory Peptide Sequence	Amino Acid Residues	pI	GRAVY *	IC_50_ (µM)	Mode of Inhibition
RIGLF	5	9.75	1.240	116	Competitive
AHEPVK	6	6.79	−1.033	63	Competitive
PSSNK	5	9.18	−2.120	129	Non-competitive
VIEKYP	6	5.97	−0.267	130	Competitive
LSMGSASLSP	10	5.52	0.61	335	Non-competitive
GQP	3	5.52	n.d.	3.2	Competitive
GQGGP	5	5.52	−1.260	254	Competitive
RLPSEFDLSAFLRA	14	6.07	0.100	52	Competitive
RLSGQTIEVTSEYLFRH	17	6.76	−0.488	1079	Non-competitive

* The grand average of hydropathicity (GRAVY) value was obtained by analyzing the peptide amino acid residues with ProtParam tool via Expasy; n.d., not determined since at least 5 amino acid residues are required for the analysis; pI, isoelectric point.

## Data Availability

The data presented in this study are available in this article.

## Abbreviations

ACE: angiotensin I-converting enzyme; ACP, anticancer peptides; AMPs, antimicrobial peptides; BPs, bioactive peptides; CULP, *C. caelata* ubiquitin-like peptide; GLP, *Ganoderma lucidum* peptide; POP, *Pleurotus ostreatus* peptide; PSULP, *Pleurotus sajor-caju* ubiquitin-like peptide.
